# Exploring the role of self-led debriefings within simulation-based education: time to challenge the status quo?

**DOI:** 10.1186/s41077-025-00342-6

**Published:** 2025-03-11

**Authors:** Prashant Kumar, Neil Malcolm Harrison, Katy McAleer, Ibraaheem Khan, Susan Geraldine Somerville

**Affiliations:** 1 Department of Medical Education, NHS Greater Glasgow & Clyde, Scotland, UK; 2 School of Medicine, Dentistry & Nursing, University of Glasgow, Scotland, UK; 3 Department of Anaesthesia, NHS Greater Glasgow & Clyde, Scotland, UK; 4 Centre for Medical Education & Dundee Institute for Healthcare Simulation, School of Medicine, University of Dundee, Scotland, UK

**Keywords:** Debriefing, Self-led debriefing, Self-debriefing, Self-guided debriefing, Peer-led debriefing, Peer-debriefing, Unfacilitated debriefing, Simulation-based education

## Abstract

**Background:**

The notion that debriefing quality is highly reliant on the skills and expertise of the facilitator is being increasingly challenged. There is therefore emerging interest in self-led debriefings (SLDs), whereby following a simulated learning event, individuals or groups of learners conduct a debriefing amongst themselves, without the immediate presence of a trained facilitator. The interest in this approach to debriefing is multifactorial but is, in part, driven by a desire to reduce costs associated with resource-intensive faculty presence. The debate regarding the role of SLDs in simulation-based education (SBE) therefore has important implications for the simulation community.

**Main body:**

We comprehensively explore the role of SLDs by contextualising their application across the spectrum of SBE, both in terms of contrasting simulation factors, namely (i) simulation modality, (ii) debriefing forum, and (iii) debriefing adjuncts, as well as different learner characteristics, namely (i) learners’ previous simulation experience, (ii) learner numbers, and (iii) learners’ professional and cultural backgrounds. These factors inherently shape the conduct and format of SLDs, and thus impact their effectiveness in influencing learning. We have synthesised and critically analysed the available literature to illuminate this discussion.

**Conclusions:**

The current evidence suggests that SLDs can, in the right circumstances, form part of an effective debriefing strategy and support learners to reach appropriate levels of critical self-reflection and learning. Careful consideration and due diligence must go into the design and implementation of SLDs to augment the advantages of this debriefing format, such as enhancing flexibility and learner autonomy, whilst mitigating potential risks, such as reinforcing errors and biases or causing psychological harm. In situations where resources for facilitator-led debriefings (FLDs) are limited, simulation educators should recognise SLDs as a potential avenue to explore in their local contexts. By leveraging the strengths of both formats, balancing learner autonomy and expert guidance, a combined SLD and FLD approach may yet prove to be the optimal debriefing strategy to maximise learning. Whilst more research is needed to deepen our understanding of the nuances of SLDs to assess their true applicability across the spectrum of SBE, the time may now have arrived to consider challenging the status quo.

## Background

Frequently cited as the most critical component for promoting learning in simulation-based education (SBE) [1–4], debriefings should support learners to deliberately reflect on actions and develop strategies for future growth within a psychologically safe environment [5]. Typically, debriefings are facilitated by trained faculty to ensure content relevance and learners’ attainment of intended learning outcomes (ILOs) [6]. Simulation experts consider debriefing quality to be highly reliant on the skills and expertise of the facilitator [1, 3, 4, 7, 8], whose key role is to enable progressivity; the notion of progressing talk during debriefings into authentic learning conversations [9]. These observations are echoed in literature from non-healthcare industries that suggest facilitators enhance reflexivity, concentration, and psychological safety, thereby leading to improved learning [10, 11]. However, this position is being increasingly challenged [12–16], with some commentators advocating for the consideration of self-led debriefings (SLDs) as an alternative to the well-established practice of facilitator-led debriefings (FLDs) [16, 17]. This is, in part, driven by a desire to reduce costs associated with SBE [18]. By reducing faculty presence, proponents of SLDs argue that they offer a cost-effective alternative to FLDs [2, 13, 14, 18–22]. Considering the significant resources required to deliver facilitator-led SBE and sustain faculty development programmes [1, 23–25], this debate has important implications for the simulation community.

SLDs are defined as “debriefings that occur without the immediate presence of a trained faculty member, such that the debriefing is conducted by the learners themselves” ( [26], p., 2). The term ‘SLDs’ is often used interchangeably with self-debriefings, self-guided debriefings, peer-debriefings, peer-led debriefings and unfacilitated debriefings. The increasing use of SLDs has been mirrored by an evolving evidence base exploring their role within SBE, and published reviews have reported little difference in debriefing outcomes between SLDs and FLDs [1, 2, 4, 6, 15, 27–32]. However, SLDs encompass a variety of heterogenous practices, and therefore such conclusions risk oversimplifying an inherently complex topic. Rather than focussing on generic comparisons between SLDs and FLDs, we advocate consideration of the potential impact contextual factors have on learning experiences, specifically in relation to SLDs. Currently, there is limited literature exploring these issues in sufficient depth.

In this debate article, we aim to explore this gap by contextualising the application of SLDs, both in terms of contrasting simulation factors, namely (i) simulation modality, (ii) debriefing forum, and (iii) debriefing adjuncts, as well as different learner characteristics, namely (i) learners’ previous simulation experience, (ii) learner numbers, and (iii) learners’ professional and cultural backgrounds. We have synthesised and critically analysed the available literature to illuminate this debate, allowing readers to consider whether SLDs have a place in their current practice as simulation educators. We then discuss the potential value of employing combined self-led and facilitator-led debriefing strategies and highlight gaps in the literature that require addressing to further inform and deepen our understanding of the role of SLDs within SBE.

## Contextual factors influencing self-led debriefings

**Fig. 1:**
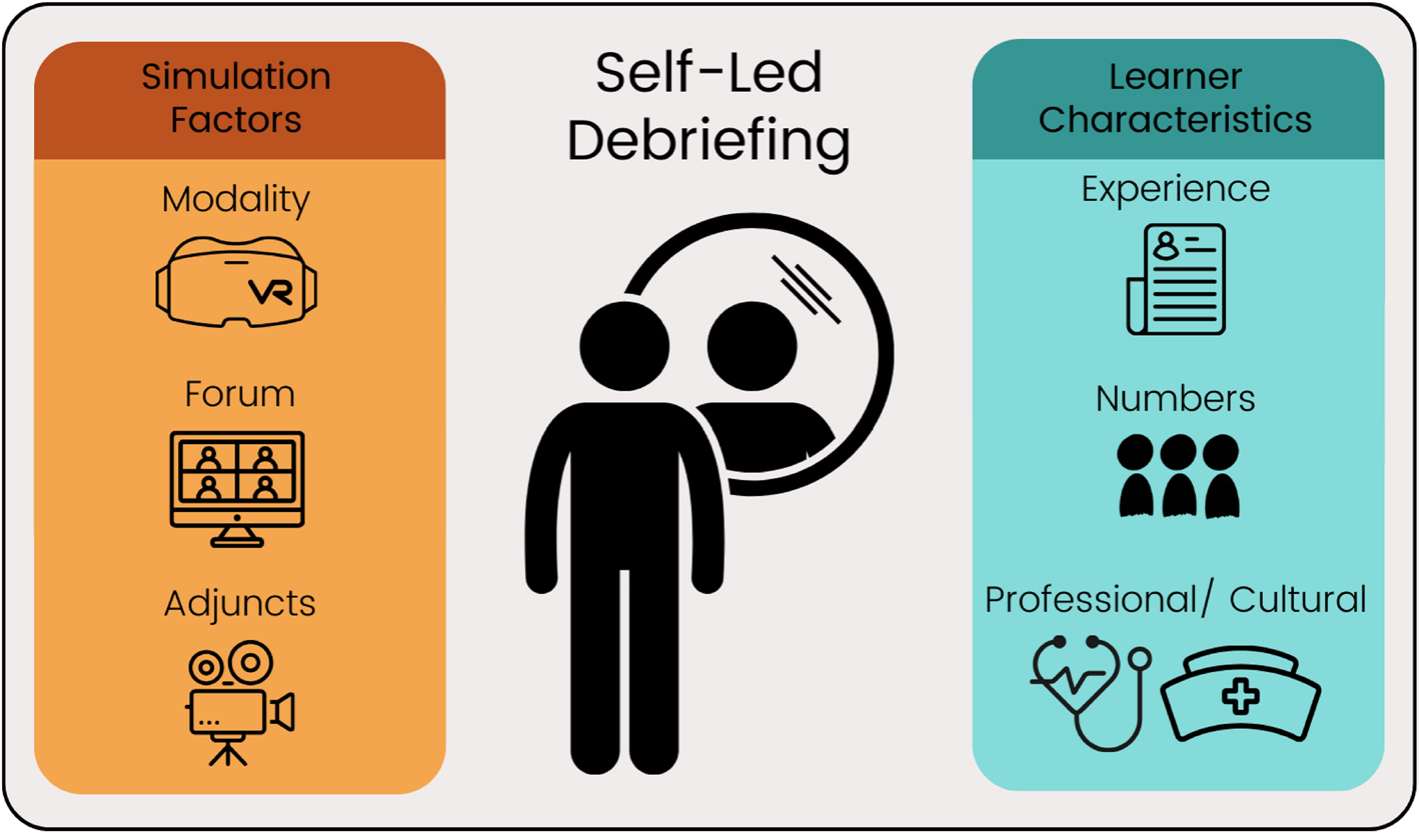
Simulation factors and learner characteristics influencing self-led debriefings

There is increasing recognition that various debriefing methods, such as SLDs, can be effectively applied in differing contexts across the spectrum of SBE practice [6]. The potential benefits and challenges of SLDs are impacted by particular simulation factors and learner characteristics (Fig. 1), and we now explore these in turn.

### Simulation factors

#### (i) Simulation modality

SBE is practiced using a variety of modalities, including part-task procedure-based simulations, fully immersive scenarios and extended reality (XR) technologies. In the following paragraphs, we discuss each of these modalities in turn.

Part-task procedure-based simulations often involve fundamentally different debriefing approaches that are centred around mastery learning [6, 9]. In such contexts, some simulation educators utilise and advocate for within-event ‘pause, correct, and repeat’ feedback and debriefing approaches such as rapid cycle deliberate practice [33–35]. Others, however, report no difference between within-event and post-event FLDs [36, 37] or suggest that post-event FLDs may be superior, especially for novice learners learning simple tasks [38]. If FLDs were replaced by SLDs, the lack of concurrent expert feedback, coaching and guidance with correction of practice, is likely to hinder learning, especially if learners are unaware of their shortcomings. The potential consolidation of inaccurate information and biases without correction will adversely impact future performance [22, 39–41] and potentially endure into the clinical workplace [42]. Despite these concerns, SLDs have been used for such contexts, with mixed results. Certain studies have demonstrated that learners undergoing SLDs can improve performance of psychomotor skills such as cardiopulmonary resuscitation quality [43], laparoscopic suturing [44], and laparoscopic robotic skills [45]. Equivalent outcomes are reported when comparing SLDs and FLDs for certain skills, including intravenous cannulation [46] and diagnosing cardiac murmurs [47]. In contrast, learners undergoing SLDs demonstrated reduced cardiopulmonary resuscitation performance [48] and lesser improvements in laparoscopic suturing skills [44], when compared to FLD groups. To mitigate the risks of reinforcing suboptimal or incorrect performance in part-task skills training contexts, we recommend learners be provided with demonstrations of best practices, either in the form of guidelines or instructional audiovisual segments.

Immersive scenario-based simulation often aims to replicate real clinical encounters, with learners working individually or in teams to assess and manage clinical scenarios. When faced with inherent complexity, both in terms of scenario execution and content of the subsequent debriefing conversations, the limitations of SLDs become more apparent [17]. Learners may find it difficult to navigate the intricate nuances of individual and team performance in dynamic and stressful clinical situations. There is a risk of learners engaging only in superficial levels of self-reflection, subsequently leading to missed opportunities to affect behaviour change [48–50]. These challenges can be mitigated by employing structured strategies such as pre-training learners in debriefing skills and methods [51, 52], using written frameworks to guide learners through the debriefing process [6, 16, 52–55], or employing online tools aimed at assisting learners to self-debrief with a critical perspective [50]. However, many commentators advocate that in such circumstances facilitator expertise remains crucial in helping learners explore critical discussion points, surface assumptions, survey contrasting perspectives, and examine complex teamwork dynamics [3, 17, 56]. Furthermore, by providing expert insight and enabling progressivity, facilitators can help ensure appropriate depth of reflection and analysis occurs, thus allowing learners to gain meaning from their simulated experiences and apply these lessons to clinical practice [3, 9]. We would therefore urge caution in applying SLDs to such contexts.

XR technologies, spanning virtual, augmented and mixed reality platforms, have been increasingly embedded into SBE practice. However, formal debriefing is commonly omitted in these settings [57], and it remains one setting in which there is little evidence exploring the role of SLDs. XR platforms offer learners the opportunity for repeated deliberate practice and to receive immediate automated performance feedback that can incorporate metrics such as motion tracking, haptic feedback, task performance accuracy, and response times [58–60]. Such performance analytics may be integrated into SLDs to support learners’ self-reflective processing during XR experiences. However, the role of expert facilitation in encouraging learners to make sense of, and formulate meaning from, such analytical data remains undetermined, with authors cautioning against XR replacing the presence and guidance of expert educators [60, 61].

#### (ii) Debriefing forum

Traditionally, debriefings in SBE are conducted in-person, with facilitators debriefing learners immediately after a simulated learning event. In recent years, distance telesimulation modalities have led to virtual debriefing forums increasing in use [62, 63]. Several studies have concluded that individual virtual SLDs can offer a practical, safe and effective forum to support learning, enhance self-awareness and promote self-reflection [55, 63–66]. Allowing for both synchronous and asynchronous debriefings, SLDs offer flexibility in the method, process, and pace of learning [2, 19, 67, 68]. Learners can independently personalise the scheduling of debriefings, adapting the pace and depth of reflection to suit their own individual learning needs [2, 67–69]. Individual virtual SLDs are typically conducted with an online written activity or guide that aims to facilitate learners’ reflections following a simulation [55, 63, 66, 70]. Verkuyl et al. [63] advocate that such SLD guides should be carefully crafted and consider contextual factors such as learner demographics, clinical experiences, and desired ILOs to truly evoke critical thinking. However, there remain concerns regarding virtual SLDs, including varying levels of learner motivation to engage with reflective questions and processes, variable depth of self-reflection evident in learners’ written accounts, and the time and resource requirements for educators to review learners’ post-event written reflective accounts [67, 70]. Furthermore, the process, practicalities and role of group virtual SLDs remain unexplored in the literature. A recent scoping review highlighted the importance of a facilitator’s role in the feedback loop during and following virtual simulations [71], commented on by students as a crucial element for learning [72, 73]. Similarly, a systematic review investigating debriefing methods for virtual simulations reported that facilitator experience and skill strongly influenced debriefing quality [74]. Nevertheless, individual virtual SLDs confer benefits to learners whilst safeguarding effective learning [55, 63–66].

Under certain conditions, in-person group SLDs can enable learners to achieve suitable levels of critical self-reflection and provide an alternative method to FLDs to safeguard effective learning [54]. Several papers have reported equivalent outcomes between groups undergoing either in-person SLDs or FLDs [12, 13, 16, 19, 68, 69, 75–77]. Across these studies, however, significant heterogeneity exists between course aims, scenario designs, SLD formats, debriefing adjuncts, learner characteristics, and outcome measures used. In a study of nursing students Gnatt et al. [49] found that not only did both learners and faculty prefer FLDs to SLDs, but that the group undergoing FLDs had significantly improved performance scores. Other studies have also demonstrated learner preference for FLDs over SLDs [48, 78, 79], indicating their need for faculty reassurance and accurate debriefing content [54]. Simulation educators similarly report preferring FLDs, stating that they provide a more “creative and constructive learning experience” ( [49], p., 13). Additional studies have reported in-person FLDs to be significantly more effective than SLDs across some, but not all, of their stated outcome measures, including learner self-confidence, debriefing quality, and reflection [40, 42, 80]. We therefore urge caution in utilising SLDs in such contexts.

#### (iii) Debriefing adjuncts

Debriefing research has often focussed on debriefing adjuncts, such as audiovisual playback and written instruments, which facilitators use to structure and guide their debriefing practice [4, 15, 28, 32]. Similar attention to these elements is now materialising within the SLD literature.

Video-assisted SLDs are commonly employed and studies report that their use contributes to enhancing self-reflection by enabling learners to analyse performance, minimise hindsight bias and identify behaviours and mannerisms which they may not have been conscious of [12–14, 44, 69, 76, 81–84]. Furthermore, comparative studies have shown equivalence in performance outcomes and learner satisfaction between video-assisted SLDs and FLDs [12, 13, 69, 76]. In the context of procedural skills training, a video recording system integrated into an SLD protocol led to a reduction in robotic surgical skill decay compared to an SLD-only group [45], whilst another suggested that incorporating audiovisual playback into SLDs improved proficiency in laparoscopic suturing skills [44]. Conversely, audiovisual playback may reduce learners’ abilities to meaningfully engage with self-reflection due to feelings of self-consciousness and anxiety [83, 85, 86], feelings that may be accentuated in group settings whereby videos of learners participating in simulation scenarios are observed by peers. In such cases, the role of facilitator expertise and skill in using, or omitting, audiovisual playback appropriately, may safeguard learning whilst preventing psychological harm [1, 84, 86]. An alternative option to mitigate this risk is to employ individual video-assisted SLDs prior to group debriefings, a strategy endorsed by some study participants [82]. Video-assisted feedback has promise to enhance SLDs but careful consideration of how they are incorporated into the debriefing process is needed [69].

Studies examining SLDs often include a written instrument for learners to document their impressions of the preceding simulation event [39–41, 53, 55, 63, 66, 67, 75, 79]. It has been suggested that written debriefings can enable the articulation and structuring of complex mental processes, thereby leading to the interpretation of events at a higher cognitive level, beyond what can be achieved through discussion alone [87–89]. However, this is highly dependent on the content, quantity, and quality of the questions that make up the instrument [66, 70]. In their study of nursing students undergoing SLDs, MacKenna et al. [66] concluded that responses to analytical-based questions yielded the highest proportion of critical reflection amongst learners, whilst evaluation and future planning-based questions yielded the lowest. Learner perceptions of written debriefing remain mixed, with some reporting that working through written checklists of expected behaviours allowed them to identify specific actions to search for and analyse [69], whilst others reported a preference for oral over written debriefing [90]. In a study of online discussion board SLDs, learners showed a lack of understanding about the purpose of debriefing and displayed no intent for self-reflection [70]. In SLD contexts, therefore, careful consideration should be given to crafting appropriate and meaningful written instruments that explicitly align with ILOs and encourage deep self-reflective thinking amongst learners [1, 63, 66].

### Learner characteristics

#### (i) Learners’ previous simulation experience

Learners’ previous simulation experience significantly impacts their ability to meaningfully engage with the reflective nature of the SLD process [13, 14, 17, 21, 54]. Learners with multiple prior experiences of FLDs are better prepared to integrate typical debriefing goals, structures, and processes whilst effectively critiquing their own actions, behaviours and performances within SLD forums [14, 20]. In a study investigating paramedic students, Christiansen et al. [21] demonstrated that this phenomenon seems to hold true even in learner cohorts with relatively little real-world clinical experience but significant prior involvement with SBE. Conversely, however, healthcare practitioners’ real-world clinical experiences allow them to recontextualise their simulated experiences more readily and in this manner act as a gateway into the reflective process [14]. Lapum et al. [67] argue that both reflection and analysis are learned activities and that learners’ abilities evolve over time, a factor that may limit novice learners’ capacity to effectively participate in SLDs. There is the potential therefore to focus on unimportant topics, reinforce erroneous information and biases, and miss learning opportunities for closing knowledge gaps [49, 50, 54, 55]. Novice learners may struggle to guide discussions and structure feedback amongst themselves, leading to cognitive overload that adversely affects their abilities to self-assess and self-reflect. They are therefore more likely to benefit from facilitator-guided debriefing and directive feedback to reduce misinterpretations and achieve their ILOs [6, 17]. Despite this, several studies have demonstrated a degree of equivalency in some debriefing outcomes between FLD and SLD groups of undergraduate students [16, 40, 42, 64, 69, 75, 80, 81, 91]. However, these studies are limited by the heterogeneity and quality of outcome measures, and we therefore advocate that SLDs are more appropriate for learners with significant prior simulation experience.

#### (ii) Learner numbers

Individual and group SLDs are fundamentally separate activities with inherently distinct challenges. They should therefore be treated as such, both in the debriefing literature as well as in practice.

An integrative review reported comparable learning outcomes between in-person and virtual individual SLDs and FLDs [20]. Individual SLDs allow learners time and space to deconstruct their experiences and formulate meaning from those experiences, without the pressure of having to respond to questions immediately [55, 92]. Furthermore, SLDs can support learner-centredness and boost learner autonomy by enhancing ownership and control of their own learning [47, 50, 66]. By encouraging personal responsibility for learning, SLDs empower learners to identify their own learning needs and goals [12, 68], possibly leading to improved engagement and motivation to self-reflect on their simulation experience [21, 42, 52]. For some learners, being observed and then having their performance discussed in group settings can be anxiety-provoking and potentially impede their learning. In such cases, individual SLDs may help reduce stress and anxiety [68, 92], offering a suitable alternative to group debriefings. Conversely, however, in individual SLDs, the potential benefits afforded by the support, insights and constructive feedback from peers is lost. Individuals may find it difficult to reflect on their own biases and performance and may benefit more from group feedback and collaborative discussion. Importantly, different learners from within the same course may benefit more from one approach over the other. How educators identify those in each group at the outset of a simulation course remains an unenviable task.

The picture is more complex for group SLDs. Kumar and Somerville’s [54] integrative review of in-person group SLDs concluded that, across a range of debriefing outcomes, whilst SLDs were not preferable to FLDs, in certain circumstances, they can enable learners to achieve suitable levels of critical self-reflection and learning. Reflexive thematic analysis of the data set demonstrated that stimulating self-reflective practice amongst learners remains the key fundamental factor of how and why group SLDs influence debriefing outcomes. In group contexts, SLDs offer opportunities for reflection on collaborative practice and teamwork dynamics both within and beyond learners’ own professional groups. The process of providing constructive feedback to peers in group SLDs potentially creates an opportunity for learners to gain better self-awareness and understanding of their own abilities, strengths and weaknesses [93]. However, the requirement of an expert facilitator to enable such knowledge sharing to occur remains undetermined. For example, in group SLDs, unequal participation amongst learners may occur with more dominant members of the group dictating conversation, potentially overlooking valuable insights from more reserved learners within the group [50]. Additionally, how learners manage conflict amongst themselves is unknown and may lead to psychological harm if not appropriately identified and attended to.

The familiarity of linking with peers in SLDs has been reported to foster psychological safety and promote learning [94], although how, why and if this is consistently achieved remains contentious. Creating and maintaining psychological safety, where learners feel safe to take interpersonal risks [95], during group debriefings is paramount to optimise learning [6, 96, 97]. Typically, the role and skill of the facilitator is thought to be key in this dynamic process [3, 97–99]. Specifically, managing learners’ emotions following participation in simulation activities, and their subsequent impact on a psychologically safe environment conducive to learning, is a challenging and daunting undertaking. Facilitator attributes and actions that help this process include displaying honesty, adaptability and flexibility, maximising authenticity, conveying a growth mindset, reading body language, using silence, modelling vulnerability and actively listening [9, 97, 100], skills that require deliberate training and coaching to develop [86, 101]. One may infer, therefore, that the challenge of fostering psychological safety in contexts without the immediate presence of trained faculty is heightened, with an associated risk of psychological harm to learners. We remain concerned that despite the importance placed on fostering psychological safety in FLDs, the same emphasis is not yet apparent in the SLD literature.

#### (iii) Learners’ professional and cultural backgrounds

Learners’ professional backgrounds may have a profound impact on the depth of reflection and learning within SLDs. Most of the current evidence base concerning SLDs stems from the nursing profession, although it is unclear why this is the case. Some commentators have advocated that in interprofessional contexts, the inherent complexity of managing diverse individual learning needs, deeply complicated group dynamics, and historical power imbalances, hierarchies and professional divisions [3, 102–104], mandates the presence of facilitator expertise [3, 105]. In an arena where facilitator skill has been the most frequently cited enabler of psychological safety [98], simulation educators may feel uncomfortable entrusting challenging learning conversations to interprofessional learners themselves, due to concerns of reinforcing stereotypes, embedding hierarchical power imbalances and accentuating the risk of psychological harm. For example, in two articles describing the same interprofessional SLD learner sample, in which facilitation was left spontaneously to the learners themselves, sixteen SLDs were noted to be physician-led whilst only one was nurse-led [13, 16]. Similarly, Ju et al. [106] describe comparable observations of physician dominance amongst interprofessional faculty within debriefings, leading to suboptimal interprofessional practice and role-modelling. If such concerns remain a challenge amongst experienced faculty, then despite some isolated studies rating the quality of interprofessional SLDs highly [107], we are likely asking too much of interprofessional learners to govern such challenges themselves.

Learners’ cultural backgrounds may also significantly influence the use and impact of SLDs because learners’ engagement with debriefing practice relies on their underlying values, beliefs and attitudes. Cultural influences play a fundamental role in how humans interact with, and learn from, one another in debriefing settings [108, 109]. In some instances, the introspection and self-awareness required for effective SLDs may align with existing cultural norms, whereas in others they may manifest as significant barriers. Different cultures possess inherently distinct attitudes towards factors like authority and hierarchy, communication etiquette, perceptions of mistakes and failures, learner autonomy, and comfort with self-reflection. Our understanding of how these differences may influence the implementation of SLDs, and indeed manifest within SLD forums themselves if cultural diversity is present amongst learners, is lacking. Whilst SLDs have been studied across diverse cultural settings, there have been no studies examining how cultural diversity actively influences the process of SLD or the potential impact it may have on learning within this forum. As simulation educators, we should therefore be cognisant of deficits of understanding within this topic [110].

## Combined approaches using self-led debriefings with facilitator-led debriefings

By leveraging the strengths of both formats, balancing learner autonomy and expert guidance, a combined SLD and FLD approach may yet prove to be the optimal debriefing strategy to maximise learning [54]. Improved outcomes for combined approaches have been reported for in-person group debriefings [22, 79, 80, 91]. These findings are supported by both quantitative and qualitative studies investigating in-person and virtual individual SLD formats combined with group FLDs, reporting improved debriefing outcomes across multiple domains including self-efficacy, self-awareness, fortifying knowledge, reflection, and learner experience [65, 67, 92, 94, 111, 112]. In these studies, individual SLD elements allowed learners the opportunity to authentically process their reactions and emotions, organise their thoughts and reflections, identify knowledge gaps, and build confidence prior to engaging in group FLDs. The group FLD elements then enabled learners to clarify any misconceptions, address unanswered questions, gain valuable insights from peers, and undertake deeper introspection than achieved in the SLDs alone. Furthermore, learners tend to prefer and value combined approaches to SLD-only approaches [65, 113]. Simulation educators also seem to recognise the benefits of this approach, noting that by allowing learners to debrief amongst themselves first, they would relax, become comfortable talking in unfamiliar groups, and make joint decisions about what they wished to discuss, thus leading to more learner-centred FLDs afterward [104]. A combined approach can bring together the inherent benefits of both SLD and FLD approaches, whilst providing some mitigation to the challenges and risks presented by SLDs. Whilst more research is required to better understand how, why and for whom this approach best serves, it appears to be well-suited for enhancing the overall learning experience.

## Recommendations for future research

Despite the increased popularity of SLDs and the corresponding increase in its evidence base, there remain several areas requiring further research. Firstly, whilst there is a justified focus on psychological safety in FLDs, the same is not currently true in SLD contexts. This issue should be explored to develop our understanding of how best to foster psychological safety whilst mitigating the inherent risks associated with SLDs to prevent psychological harm. Secondly, there remains a huge gap attesting to the role of SLDs in XR platforms within SBE, in particular how learners may best incorporate automated performance feedback into their self-reflective processes. Thirdly, the role of combined SLD and FLD approaches should be researched, both quantitatively and qualitatively, to assess how and why different aspects of the process, format, and timing may affect learning. Finally, more research is needed to investigate how and why learners may learn differently, in terms of their mental and cognitive processing, when a facilitator is present compared with when they are alone or amongst peers. Answers to such questions may provide more nuanced insights into the contextualisation of SLDs across varying simulation factors and learner characteristics, such that simulation educators, and indeed learners themselves, will be better placed to judge what, if any, role SLDs hold in their simulation practice.

## Conclusions

There are various methods of debriefing from which simulation educators can choose. SLDs are one such method. In this article we have comprehensively explored the role of SLDs in SBE and contextualised their application across a variety of simulation factors and set of learner characteristics, debating both their benefits and challenges across this spectrum. The current evidence suggests that SLDs can, in the right circumstances, form part of an effective debriefing strategy and support learners to reach appropriate levels of critical self-reflection and learning. Careful consideration and due diligence must go into the design and implementation of SLDs to augment the advantages of this debriefing format, such as enhancing flexibility and learner autonomy, whilst mitigating any potential risks, such as reinforcing errors and biases or causing psychological harm. As such, in situations where resources for FLDs are limited, simulation educators should recognise SLDs as a potential avenue to explore in their local contexts. By leveraging the strengths of both formats, balancing learner autonomy and expert guidance, a combined SLD and FLD approach may yet prove to be the optimal debriefing strategy to maximise learning. Whilst more research is needed to deepen our understanding of the nuances of SLDs to assess their true applicability across the spectrum of SBE, the time may now have arrived to consider challenging the status quo.

## Data Availability

No datasets were generated or analysed during the current study.

## References

[1] Fanning RM, Gaba DM. The role of debriefing in simulation-based learning. Simul Healthc. 2007;2(2):115–25. 19088616 10.1097/SIH.0b013e3180315539

[2] Levett-Jones T, Lapkin S. A systematic review of the effectiveness of simulation debriefing in health professional education. Nurse Educ Today. 2014;34(6):e58–63. 24169444 10.1016/j.nedt.2013.09.020

[3] Kumar P, Paton C, Simpson HM, King CM, McGowan N. Is interprofessional co-debriefing necessary for effective interprofessional learning within simulation-based education? IJoHS. 2021;1(1):49–55.

[4] Endacott R, Gale T, O’Connor A, Dix S. Frameworks and quality measures used for debriefing in team-based simulation: a systematic review. BMJ Simul Technol Enhanc Learn. 2019;5(2):61–72. 35519834 10.1136/bmjstel-2017-000297 PMC8936997

[5] Cheng A, Morse KJ, Rudolph J, Arab AA, Runnacles J, Eppich W. Learner-centered debriefing for health care simulation education: lessons for faculty development. Simul Healthc. 2016;11(1):32–40. 26836466 10.1097/SIH.0000000000000136

[6] Sawyer T, Eppich W, Brett-Fleegler M, Grant V, Cheng A. More than one way to debrief: a critical review of healthcare simulation debriefing methods. Simul Healthc. 2016;11(3):209–17. 27254527 10.1097/SIH.0000000000000148

[7] Cheng A, Grant V, Huffman J, Burgess G, Szyld D, Robinson T, et al. Coaching the debriefer: peer coaching to improve debriefing quality in simulation programs. Simul Healthc. 2017;12(5):319–25. 28538446 10.1097/SIH.0000000000000232

[8] Paige JT, Arora S, Fernandez G, Seymour N. Debriefing 101: training faculty to promote learning in simulation-based training. Am J Surg. 2015;209(1):126–31. 25497438 10.1016/j.amjsurg.2014.05.034

[9] Kainth R, Reedy G. Transforming professional identity in simulation debriefing: a systematic metaethnographic synthesis of the simulation literature. Simul Healthc. 2024;19(2):90–104. 37335122 10.1097/SIH.0000000000000734

[10] Allen JA, Reiter-Palmon R, Crowe J, Scott C. Debriefs: teams learning from doing in context. Am Psychol. 2018;73(4):504–16. 29792464 10.1037/amp0000246

[11] Tannenbaum SI, Cerasoli CP. Do team and individual debriefs enhance performance? A meta-analysis. Hum Factors. 2013;55(1):231–45. 23516804 10.1177/0018720812448394

[12] Boet S, Bould MD, Bruppacher HR, Desjardins F, Chandra DB, Naik VN. Looking in the mirror: self-debriefing versus instructor debriefing for simulated crises. Crit Care Med. 2011;39(6):1377–81. 21317645 10.1097/CCM.0b013e31820eb8be

[13] Boet S, Bould MD, Sharma B, Reeves S, Naik VN, Triby E, et al. Within-team debriefing versus instructor-led debriefing for simulation-based education: a randomized controlled trial. Ann Surg. 2013;258(1):53–8. 23728281 10.1097/SLA.0b013e31829659e4

[14] Boet S, Pigford A, Fitzsimmons A, Reeves S, Triby E, Bould MD. Interprofessional team debriefings with or without an instructor after a simulated crisis scenario: an exploratory case study. J Interprof Care. 2016;30(6):717–25. 27309589 10.1080/13561820.2016.1181616

[15] Garden AL, Le Fevre DM, Waddington HL, Weller JM. Debriefing after simulation-based non-technical skill training in healthcare: a systematic review of effective practice. Anaesth Intensive Care. 2015;43(3):300–8. 25943601 10.1177/0310057X1504300303

[16] Jaffrelot M, Boet S, Floch Y, Garg N, Dubois D, Laparra V, et al. Learning with our peers: peer-led versus instructor-led debriefing for simulated crises, a randomized controlled trial. Korean J Anesthesiol. 2024;77(2):265–72. 38556779 10.4097/kja.23317 PMC10982526

[17] Harder N, Turner S, Kramer M, Mitchell K. Exploring debriefing modalities in healthcare simulation: self-reflection, self-debriefing, tele-debriefing and facilitated debriefing. Clin Sim Nurs. 2024;92: 101561.

[18] Isaranuwatchai W, Alam F, Hoch J, Boet S. A cost-effectiveness analysis of self-debriefing versus instructor debriefing for simulated crises in perioperative medicine in Canada. J Educ Eval Health Prof. 2016;13: 44. 28028288 10.3352/jeehp.2016.13.44 PMC5286203

[19] Devine LA, Donkers J, Brydges R, Perelman V, Cavalcanti RB, Issenberg SB. An equivalence trial comparing instructor-regulated with directed self-regulated mastery learning of advanced cardiac life support skills. Simul Healthc. 2015;10(4):202–9. 26154249 10.1097/SIH.0000000000000095

[20] MacKenna V, Díaz DA, Chase SK, Boden CJ, Loerzel V. Self-debriefing in healthcare simulation: an integrative literature review. Nurse Educ Today. 2021;102: 104907. 33901867 10.1016/j.nedt.2021.104907

[21] Christiansen CR, Anderson JV, Dieckmann P. Comparing reflection levels between facilitator-led and student-led debriefing in simulation training for paramedic students. Adv Simul. 2023;8(1):30. 10.1186/s41077-023-00273-0 PMC10722852 38098131

[22] Kang K, Yu M. Comparison of student self-debriefing versus instructor debriefing in nursing simulation: a quasi-experimental study. Nurse Educ Today. 2018;65:67–73. 29533836 10.1016/j.nedt.2018.02.030

[23] Kumar P, Collins K, Paton C, McGowan N. Continuing professional development for faculty in simulation-based education. IJoHS. 2021;1(1):63.

[24] Chung HS, Issenberg SB, Phrampus P, Miller G, Je SM, Lim TH, et al. International collaborative faculty development program on simulation-based healthcare education: a report on its successes and challenges. Korean J Med Educ. 2012;24(4):319–27. 25813328 10.3946/kjme.2012.24.4.319 PMC8813360

[25] Gardner AK, Rodgers DL, Steinert Y, Davis R, Condron C, Peterson DT, et al. Mapping the terrain of faculty development for simulation: a scoping review. Simul Healthc. 2024;19(1S):S75–89. 38240621 10.1097/SIH.0000000000000758

[26] Kumar P, Somerville S. Exploring self-led debriefings in simulation-based education: an integrative review protocol. IJoHS. 2023;1–10. https://www.ijohs.com/article/doi/10.54531/fxbh1520. 10.1186/s41077-023-00274-z PMC10790376 38229166

[27] Cheng A, Eppich W, Grant V, Sherbino J, Zendejas B, Cook DA. Debriefing for technology-enhanced simulation: a systematic review and meta-analysis. Med Educ. 2014;48:657–66. 24909527 10.1111/medu.12432

[28] Dufrene C, Young A. Successful debriefing- best methods to achieve positive learning outcomes: a literature review. Nurse Educ Today. 2014;34(3):372–6. 23890542 10.1016/j.nedt.2013.06.026

[29] Kim Y, Yoo J. The utilization of debriefing for simulation in healthcare: a literature review. Nurse Educ Pract. 2020;43: 102698. 32004851 10.1016/j.nepr.2020.102698

[30] Lee J, Lee H, Kim S, Choi M, Ko IS, Bae J, et al. Debriefing methods and learning outcomes in simulation nursing education: a systematic review and meta-analysis. Nurse Educ Today. 2020;87: 104345. 32135455 10.1016/j.nedt.2020.104345

[31] Niu Y, Liu T, Li K, Sun M, Sun Y, Wang X, et al. Effectiveness of simulation debriefing methods in nursing education: a systematic review and meta-analysis. Nurse Educ Today. 2021;107:105113. 34492539 10.1016/j.nedt.2021.105113

[32] Duff JP, Morse KJ, Seelandt J, Gross IT, Lydston M, Sargeant J, et al. Debriefing methods for simulation in healthcare: a systematic review. Simul Healthc. 2024;19:S112–21. 38240623 10.1097/SIH.0000000000000765

[33] Lemke DS, Young AL, Won SK, Rus MC, Villareal NN, Camp EA, et al. Rapid-cycle deliberate practice improves time to defibrillation and reduces workload: a randomized controlled trial of simulation-based education. AEM Educ Train. 2021;5(4): e10702. 34901686 10.1002/aet2.10702 PMC8637872

[34] Won SK, Doughty CB, Young AL, Welch-Horan TB, Rus MC, Camp EA, et al. Rapid cycle deliberate practice improves retention of pediatric resuscitation skills compared with postsimulation debriefing. Simul Healthc. 2022;17(1):e20–7. 34009907 10.1097/SIH.0000000000000568

[35] Eppich WJ, Hunt EA, Duval-Arnould JM, Siddall VJ, Cheng A. Structuring feedback and debriefing to achieve mastery learning goals. Acad Med. 2015;90(11):1501–8. 26375272 10.1097/ACM.0000000000000934

[36] Rosman SL, Nyirasafari R, Bwiza HM, Umuhoza C, Camp EA, Weiner DL, et al. Rapid cycle deliberate practice vs. traditional simulation in a resource-limited setting. BMC Med Educ. 2019;19(1):314. 31438936 10.1186/s12909-019-1742-4 PMC6704559

[37] Surapa Raju S, Tofil NM, Gaither SL, Norwood C, Zinkan JL, Godsey V, et al. The impact of a 9-month booster training using rapid cycle deliberate practice on pediatric resident PALS skills. Simul Healthc. 2021;16(6):e168–75. 33370083 10.1097/SIH.0000000000000538

[38] Hatala R, Cook DA, Zendejas B, Hamstra SJ, Brydges R. Feedback for simulation-based procedural skills training: a meta-analysis and critical narrative synthesis. Adv Health Sci Educ Theory Pract. 2014;19(2):251–72. 23712700 10.1007/s10459-013-9462-8

[39] Ha E. Effects of peer-led debriefing using simulation with case-based learning: written vs. observed debriefing. Nurse Educ Today. 2020;84: 104249. 31683133 10.1016/j.nedt.2019.104249

[40] Ha E, Lim EJ. Peer-led written debriefing versus instructor-led oral debriefing: using multimode simulation. Clin Simul Nurs. 2018;18:38–46.

[41] Kündig P, Tschan F, Semmer NK, Morgenthaler C, Zimmerman J, Holzer E, et al. More than experience: a post-task reflection intervention among team members enhances performance in student teams confronted with a simulated resuscitation task- a prospective randomised trial. BMJ Simul Technol Enhanc Learn. 2020;6(2):81–6. 35516080 10.1136/bmjstel-2018-000395 PMC8936849

[42] Kim SS, De Gagne JC. Instructor-led vs. peer-led debriefing in preoperative care simulation using standardized patients. Nurse Educ Today. 2018;71:34–9. 30218850 10.1016/j.nedt.2018.09.001

[43] Fan H-J, You S-H, Huang C-H, Seak C-J, Ng C-J, Li W-C, et al. Effectiveness of hands-on cardiopulmonary resuscitation practice with self-debriefing for healthcare providers: a simulation-based controlled trial. HKJEM. 2017;24(6):268–74.

[44] Halim J, Jelley J, Zhang N, Ornstein M, Patel B. The effect of verbal feedback, video feedback, and self-assessment on laparoscopic intracorporeal suturing skills in novices: a randomized trial. Surg Endosc. 2021;35(7):3787–95. 32804266 10.1007/s00464-020-07871-3

[45] Kun Y, Hubert J, Bin L, Huan WX. Self-debriefing model based on an integrated video-capture system: an efficient solution to skill degradation. J Surg Educ. 2019;76(2):362–9. 30292454 10.1016/j.jsurg.2018.08.017

[46] Rammell J, Matthan J, Gray M, Bookless LR, Nesbitt CI, Rodham P, et al. Asynchronous unsupervised video-enhanced feedback as effective as direct expert feedback in the long-term retention of practical clinical skills: randomised trial comparing 2 feedback methods in a cohort of novice medical students. J Surg Educ. 2018;75(6):1463–70. 29748142 10.1016/j.jsurg.2018.03.013

[47] Lorello GR, Hodwitz K, Issenberg SB, Brydges R. Relinquishing control? Supervisor co-regulation may disrupt students’ self-regulated learning during simulation-based training. Adv Health Sci Educ Theory Pract. 2024;29(1):9–25. 37245197 10.1007/s10459-023-10244-9

[48] Roh YS, Kelly M, Ha EH. Comparison of instructor-led versus peer-led debriefing in nursing students. Nurs Health Sci. 2016;18(2):238–45. 26833934 10.1111/nhs.12259

[49] Gantt L, Overton S, Avery J, Swanson M, Elhammoumi C. Comparison of debriefing methods and learning outcomes in human patient simulation. Clin Simul Nurs. 2018;17:7–13.

[50] Eddy ER, Tannenbaum SI, Mathieu JE. Helping teams to help themselves: comparing two team-led debriefing methods. Pers Psychol. 2013;66(4):975–1008.

[51] Dennis D, Furness A, Brosky J, Owens J, Mackintosh S. Can student-peers teach using simulated-based learning as well as faculty: a non-equivalent posttest-only study. Nurse Educ Today. 2020;91: 104470. 32454315 10.1016/j.nedt.2020.104470

[52] Leigh GT, Miller LB, Ardoin KB. A nurse educator’s guide to student-led debriefing. Teach Learn Nurs. 2017;12(4):309–11.

[53] Oikawa S, Berg B, Turban J, Vincent D, Mandai Y, Birkmire-Peters D. Self-debriefing vs instructor debriefing in a pre-internship simulation curriculum: night on call. Hawaii J Med Public Health. 2016;75(5):127–32. 27239391 PMC4872264

[54] Kumar P, Somerville S. Exploring in-person self-led debriefings for groups of learners in simulation-based education: an integrative review. Adv Simul. 2024;9(1):5. 10.1186/s41077-023-00274-z PMC10790376 38229166

[55] Verkuyl M, Lapum JL, Hughes M, McCulloch T, Liu L, Mastrilli P, et al. Virtual gaming simulation: exploring self-debriefing, virtual debriefing and in-person debriefing. Clin Sim Nurs. 2018;20:7–14.

[56] Kolbe M, Grande B, Lehmann-Willenbrock N, Seelandt JC. Helping healthcare teams to debrief effectively: associations of debriefers’ actions and participants’ reflections during team debriefings. BMJ Qual Saf. 2023;32(3):160–72. 35902231 10.1136/bmjqs-2021-014393

[57] Foronda CL, Gonzalez L, Meese MM, Slamon N, Baluyot M, Lee J, et al. A comparison of virtual reality to traditional simulation in health professions education: a systematic review. Simul Healthc. 2024;19(1S):S90–7. 37651101 10.1097/SIH.0000000000000745

[58] Herur-Raman A, Almeida ND, Greenleaf W, Williams D, Karshenas A, Sherman JH. Next-generation simulation- integrating extended reality technology into medical education. Front Virtual Real. 2021;2: 693399.

[59] Dubin AK, Smith R, Julian D, Tanaka A, Mattingly P. A comparison of robotic simulation performance on basic virtual reality skills: simulator subjective versus objective assessment tools. J Minimally Invasive Gynecol. 2017;24(7):1184–9. 10.1016/j.jmig.2017.07.019 28757439

[60] Pottle J. Virtual reality and the transformation of medical education. Future Healthc J. 2019;6(3):181–5. 31660522 10.7861/fhj.2019-0036 PMC6798020

[61] Moro C, Štromberga Z, Raikos A, Stirling A. The effectiveness of virtual and augmented reality in health sciences and medical anatomy. Anat Sci Educ. 2017;10(6):549–59. 28419750 10.1002/ase.1696

[62] Bajwa M, Ahmed R, Lababidi H, Morris M, Morton A, Mosher C, et al. Development of distance simulation educator guidelines in healthcare: a delphi method application. Simul Healthc. 2024;19(1):1–10. 36598821 10.1097/SIH.0000000000000707

[63] Verkuyl M, MacKenna V, St-Amant O. Using self-debrief after a virtual simulation: the process. Clin Simul Nurs. 2021;57:48–52.

[64] Verkuyl M, Atack L, McCulloch T, Liu L, Betts L, Lapum JL, et al. Comparison of debriefing methods after a virtual simulation: an experiment. Clin Sim Nurs. 2018;19:1–7.

[65] Verkuyl M, Hughes M, Atack L, McCulloch T, Lapum JL, Romaniuk D, et al. Comparison of self-debriefing alone or in combination with group debrief. Clin Simul Nurs. 2019;37:32–9.

[66] MacKenna V, Díaz DA, Chase SK, Boden CJ, Loerzel V. Self-debriefing after virtual simulation: measuring depth of reflection. Clin Sim Nurs. 2021;52:59–67.

[67] Lapum JL, Verkuyl M, Hughes M, Romaniuk D, McCulloch T, Mastrilli P. Self-debriefing in virtual simulation. Nurse Educ. 2019;44(6):E6–8. 30585886 10.1097/NNE.0000000000000639

[68] Welke TM, LeBlanc VR, Savoldelli GL, Joo HS, Chandra DB, Crabtree NA, et al. Personalized oral debriefing versus standardized multimedia instruction after patient crisis simulation. Anesth Analg. 2009;109(1):183–9. 19535709 10.1213/ane.0b013e3181a324ab

[69] Wilbanks BA, McMullan S, Watts PI, White T, Moss J. Comparison of video-facilitated reflective practice and faculty-led debriefings. Clin Simul Nurs. 2020;42:1–7.

[70] Miller ET, Farra S, Simon A. Asynchronous online debriefing with health care workers: lessons learned. Clin Sim Nurs. 2018;20:38–45.

[71] Heyn LG, Brembo EA, Byermoen KR, Cruaud C, Eide H, Flo J, et al. Exploring facilitation in virtual simulation in nursing education: a scoping review. PEC Innov. 2023;3: 100233. 38033419 10.1016/j.pecinn.2023.100233 PMC10687044

[72] Johnsen HM, Briseid HS, Brodtkorb K, Slettebø Å, Fossum M. Nursing students’ perceptions of combining hands-on simulation with simulated patients and a serious game in preparing for clinical placement in home healthcare: a qualitative study. Nurse Educ Today. 2021;97: 104675. 33302184 10.1016/j.nedt.2020.104675

[73] Liaw SY, Choo Y, Wu LT, Lim WS, Choo H, Lim SM, et al. Wow, woo, win- healthcare students’ and facilitators’ experiences of interprofessional simulation in three-dimensional virtual world: a qualitative evaluation study. Nurse Educ Today. 2021;105: 105018. 34175564 10.1016/j.nedt.2021.105018

[74] Luctkar-Flude M, Tyerman J, Verkuyl M, Goldsworthy S, Harder N, Wilson-Keates B, et al. Effectiveness of debriefing methods for virtual simulation: a systematic review. Clin Sim Nurs. 2021;57:18–30.

[75] Na YH, Roh YS. Effects of peer-led debriefing on cognitive load, achievement emotions, and nursing performance. Clin Simul Nurs. 2021;55:1–9.

[76] Tudor GJ, Podolej GS, Willemsen-Dunlap A, Lau V, Svendsen JD, McGarvey J, et al. The equivalence of video self-review versus debriefing after simulation: can faculty resources be reallocated? AEM Educ Train. 2019;4(1):36–42. 31989069 10.1002/aet2.10372 PMC6965677

[77] Sukalich S, Elliott JO, Ruffner G. Teaching medical error disclosure to residents using patient-centred simulation training. Acad Med. 2014;89(1):136–43. 24280843 10.1097/ACM.0000000000000046

[78] Andrews E, Dickter DN, Stielstra S, Pape G, Aston SJ. Comparison of dental students’ perceived value of faculty vs. peer feedback on non-technical clinical competency assessments. J Dent Educ. 2019;83(5):536–45. 30804169 10.21815/JDE.019.056

[79] Rueda-Medina B, Schmidt-RíoValle J, González-Jiménez E, Fernández-Aparicio Á, Aguilar-Ferrándiz ME, Correa-Rodríguez M. Peer debriefing versus instructor-led debriefing for nursing simulation. J Nurs Educ. 2021;60(2):90–5. 33528579 10.3928/01484834-20210120-06

[80] Tutticci N, Coyer F, Lewis PA, Ryan M. Student facilitation of simulation debrief: measuring reflective thinking and self-efficacy. Teach Learn Nurs. 2017;12(2):128–35.

[81] Lee M, Kim S, Kang K, Kim S. Comparing the learning effects of debriefing modalities for the care of premature infants. Nurs Health Sci. 2020;22:243–53. 31793162 10.1111/nhs.12662

[82] Bussard ME. Self-reflection of video-recorded high-fidelity simulations and development of clinical judgement. J Nurs Educ. 2016;55(9):522–7. 27560120 10.3928/01484834-20160816-06

[83] Ha E. Attitudes toward video-assisted debriefing after simulation in undergraduate nursing students: an application of Q methodology. Nurse Educ Today. 2014;34(6):978–84. 24467864 10.1016/j.nedt.2014.01.003

[84] Zhang H, Mörelius E, Goh SHL, Wang W. Effectiveness of video-assisted debriefing in simulation-based health professions education: a systematic review of quantitative evidence. Nurse Educ. 2019;44(3):E1–6. 30015683 10.1097/NNE.0000000000000562

[85] Nilsen S, Baerheim A. Feedback on video recorded consultations in medical teaching: why students loathe and love it- a focus-group based qualitative study. BMC Med Educ. 2005;5: 28. 16029509 10.1186/1472-6920-5-28 PMC1190180

[86] Kumar P, Collins K, Oliver N, Duys R, Park-Ross JF, Paton C, et al. Exploring the meta-debrief: developing a toolbox for debriefing the debrief. Simul Healthc. 2024. 10.1097/SIH.0000000000000830. Online ahead of print. 39432489 10.1097/SIH.0000000000000830 PMC12129385

[87] Petranek CF. Written debriefing: the next vital step in learning with simulations. S&G. 2000;31(1):108–18.

[88] van der Meij H, Leemkuil H, Li J-L. Does individual or collaborative self-debriefing better enhance learning from games? Comput Hum Behav. 2013;29(6):2471–9.

[89] Oertig M. Debriefing in Moodle: written feedback on trust and knowledge sharing in a social dilemma game. S&G. 2010;41(3):374–89.

[90] Reed SJ. Written debriefing: evaluating the impact of the addition of a written component when debriefing simulations. Nurse Educ Pract. 2015;15(6):543–8. 26299701 10.1016/j.nepr.2015.07.011

[91] Rueda-Medina B, Gómez-Urquiza JL, Molina-Rivas E, Tapia-Haro R, Aguilar-Ferrándiz ME, Correa-Rodríguez M. A combination of self-debriefing and instructor-led debriefing improves team effectiveness in health science students. Nurse Educ. 2021;46(1):E7–11. 32433378 10.1097/NNE.0000000000000845

[92] Verkuyl M, Richie S, Cahuas D, Rowland C, Ndondo M, Larcina T, et al. Exploring self-debriefing plus group-debriefing: a focus group study. Clin Simul Nurs. 2020;43:3–9.

[93] Kim-Godwin YS, Livsey KR, Ezzell D, Highsmith C, Winslow H, Aikman AN. Students like peer evaluation during home visit simulation experiences. Clin Sim Nurs. 2013;9(11):e535–42.

[94] Verkuyl M, Lapum JL, St-Amant O, Hughes M, Romaniuk D, McCulloch T. Exploring debriefing combinations after a virtual simulation. Clin Simul Nurs. 2020;40:36–42.

[95] Edmondson A. Psychological safety and learning behaviour in work teams. Adm Sci Q. 1999;44(2):350–83.

[96] Rudolph JW, Raemer DB, Simon R. Establishing a safe container for learning in simulation: the role of the presimulation briefing. Simul Healthc. 2014;9(6):339–49. 25188485 10.1097/SIH.0000000000000047

[97] Turner S, Harder N. Psychological safe environment: a concept analysis. Clin Sim Nurs. 2018;18:47–55.

[98] Lackie K, Hayward K, Ayn C, Stilwel P, Lane J, Andrews C, et al. Creating psychological safety in interprofessional simulation for health professional learners: a scoping review of the barriers and enablers. J Interprof Care. 2023;37(2):187–202. 35403551 10.1080/13561820.2022.2052269

[99] Kostovich CT, O’Rourke J, Stephen L. Establishing psychological safety in simulation: faculty perspectives. Nurse Educ Today. 2020;91:104468. 32454316 10.1016/j.nedt.2020.104468

[100] des Ordons ALR, Eppich W, Lockyer J, Wilkie RD, Grant V, Cheng A. Guiding, Intermediating, Facilitating, and Teaching (GIFT): a conceptual framework for simulation educator roles in healthcare debriefing. Simul Healthc. 2022;17(5):283–92. 34839303 10.1097/SIH.0000000000000619

[101] Cheng A, Eppich W, Kolbe M, Meguerdichian M, Bajaj K, Grant V. A conceptual framework for the development of debriefing skills: a journey of discovery, growth, and maturity. Simul Healthc. 2020;15(1):55–60. 31743312 10.1097/SIH.0000000000000398

[102] Bunderson JS, Reagans RE. Power, status, and learning in organizations. Organ Sci. 2011;22(5):1182–94.

[103] Palaganas JC, Epps C, Raemer DB. A history of simulation-enhanced interprofessional education. J Interprof Care. 2014;28(2):110–5. 24372044 10.3109/13561820.2013.869198

[104] Holmes C, Mellanby E. Debriefing strategies for interprofessional simulation- a qualitative study. Adv Simul. 2022;7(1):18. 10.1186/s41077-022-00214-3 PMC9206121 35717254

[105] Nunnink L. Time to fire the sim educators? Not quite yet. Crit Care Med. 2011;39(6):1574–5. 21610631 10.1097/CCM.0b013e318211fa47

[106] Ju M, Bochatay N, Werne A, Essakow J, Tsang L, Nottingham M, et al. Changing the conversation: impact of guidelines designed to optimize interprofessional facilitation of simulation-based team training. Adv Simul. 2024;9(1):43. 10.1186/s41077-024-00313-3 PMC11476600 39394595

[107] Paige JT, Kerdolff KE, Roger CL, Garbee DD, Yu Q, Cao W, et al. Improvement in student-led debriefing analysis after simulation-based team training using a revised teamwork assessment tool. Surgery. 2021;170(6):1659–64. 34330538 10.1016/j.surg.2021.06.014

[108] Chung HS, Dieckmann P, Issenberg SB. It is time to consider cultural differences in debriefing. Simul Healthc. 2013;8(3):166–70. 23702587 10.1097/SIH.0b013e318291d9ef

[109] Ulmer FF, Sharara-Chami R, Lakissian Z, Stocker M, Scott E, Dieckmann P. Cultural prototypes and differences in simulation debriefing. Simul Healthc. 2018;13(4):239–46. 29672469 10.1097/SIH.0000000000000320

[110] Palaganas JC, Chan AKM, Leighton K. Cultural considerations in debriefing. Simul Healthc. 2021;16(6):407–13. 34009910 10.1097/SIH.0000000000000558

[111] Verkuyl M, Atack L, Larcina T, Mack K, Cahus D, Rowland C, et al. Adding self-debrief to an in-person simulation: a mixed-methods study. Clin Simul Nurs. 2020;47:32–9.

[112] Verkuyl M, St-Amant O, Hughes M, Lapum JL, McCulloch T. Combing self-debriefing and group debriefing in simulation. Clin Sim Nurs. 2020;39:41–4.

[113] Casler K, Bobek H, Pittman O, Tornwall J. The effect of asynchronous group discussions on nurse practitioner student debriefing experience in virtual simulation. J Am Assoc Nurse Pract. 2022;34(7):901–8. 35452028 10.1097/JXX.0000000000000720

